# Platelet-rich plasma for treatment of knee osteoarthritis: a narrative review

**DOI:** 10.3389/fpain.2026.1791542

**Published:** 2026-03-09

**Authors:** Samuel L. Patton, Kelsey Gustafson, Matt Castinado, Usman Latif, Dawood Sayed, Talal W. Khan, Christopher M. Lam

**Affiliations:** Department of Anesthesiology, Pain, and Perioperative Medicine, The University of Kansas Health System, Kansas City, KS, United States

**Keywords:** interventional pain, knee osteoarthritis, pain management, platelet-rich plasma (PRP), regenerative medicine

## Abstract

Osteoarthritis is the most common cause of arthritis, a pervasive condition affecting various joints within the human body. The culmination of various factors resulting in degradation and damage of healthy cartilage initiates the cascade of degenerative changes which contribute to the development of this condition, affecting about 73% of all adults older than age 55. Various treatments are available to help treat this condition. Prevention often includes the utilization of physical therapy, exercise, and weight management while treatment of the resultant joint pain includes bracing, medications, and interventional treatments including steroid and visco supplementation techniques. Within the past two decades, particular interest has developed in utilizing regenerative methods for treatment of these degenerative conditions. One such treatment is platelet rich plasma. Here we review the salient conceptions of this treatment and provide a brief overview of the evidence and healthcare impact regarding its utilization.

## 1 Introduction

Knee pain associated with osteoarthritis, cartilage degeneration, and soft-tissue injury is one of the leading causes of disability worldwide, prompting increasing interest in minimally invasive therapies that can slow degeneration and improve function (1). Among these, platelet-rich plasma (PRP) has emerged as one of the most widely used orthobiologic treatments, driven by its autologous nature and theoretical potential to enhance tissue repair through concentrated growth factors and cytokines (2). Animal model studies suggest that localized PRP may increase healing rates in mice with simulated muscle tear injuries while *in vivo* human studies suggest intradiscal PRP may delay development and progression of intervertebral disc degeneration (3, 4).

Despite its growing popularity in sports medicine and interventional pain practices, the evidence supporting PRP remains heterogeneous, with considerable variability in preparation techniques, platelet concentrations, and clinical protocols (5). As a result, clinicians and patients are often uncertain regarding the indications for and when certain preparations of PRP is indicated. Further, equally daunting is understanding how PRP outcomes compare to other established non-surgical treatments such as corticosteroid or hyaluronic acid injections.

This paper aims to review the biological rationale, preparation methods, and current clinical evidence surrounding PRP injections for knee pathology. With a focus on clarifying its role in contemporary musculoskeletal care and identifying gaps in knowledge, We hope to highlight opportunities for future formalized research.

## 2 Biological rationale and mechanism of action

### 2.1 Composition of PRP

As its name implies, PRP is a derivative of peripheral blood processed to increase the platelet concentration above physiologic levels. PRP has also been referred to in the literature as platelet-rich growth factors, platelet-rich fibrin matrix, or platelet concentrate. In addition to platelets, this biologic also contains a wide variety of bioactive molecules including high levels of clotting factors, growth factors, cytokines, and plasma proteins with lower levels of white blood cells, red blood cells, and stem cells. The exact composition of PRP is widely variable amongst patients based on several factors including age, sex, health conditions, and hydration status (6).

Depending on the intended application, different preparation techniques can be utilized to alter the final composition. The three main sub-groups of PRP are leukocyte-rich (LR) PRP, leukocyte-poor (LP) PRP, and pure platelet-rich fibrin (P-PRF), with different uses for each preparation. Increasing the number of white blood cells contained in the injectate can augment the inflammatory process thereby increasing the speed of healing. However, this may lead to an increase in localized levels of inflammation resulting in a catabolic process. Alternatively, LP-PRP has an increased anabolic effect compared to LR-PRP but may potentially lead to excessive scar formation (7). Thus, some researchers suggest LR-PRP may have a stronger role in healing acute injuries whereas LP-PRP may be better utilized in chronic injuries, but additional studies need to be performed to further investigate this topic (8).

### 2.2 Growth factors involved

Platelets contain a wide variety of growth factors contained within granules. These granules can be categorized into three main groups including alpha, dense, and lysosomal granules together containing hundreds of types of cytokines and signaling molecules important in modifying the inflammatory cascade and tissue microenvironment. Alpha-granules are the most abundant and contain a majority of the bioactive molecules thought to be associated with PRP's efficacy. These granules contain a variety of growth factors including platelet-derived growth factor (PDGF), fibroblastic growth factor (FGF), insulin-like growth factor (IGF-1), vascular endothelial growth factor (VEGF), and transforming growth factor (TGF) amongst others in addition to chemokines and cytokines. Together, this combination of biological factors can encourage not only cell proliferation and signal cell migration but modulate inflammatory molecules as well (5). In fact, PRP has been implicated in having an antimicrobial and antifungal property due to the high level of kinocidin and reactive oxygen species release (8).

Dense granules are named for their electron-dense core and contain ATP, serotonin, dopamine, histamines, and epinephrine. These chemical messengers modify platelet activation and thrombus formation, playing a key role in coagulation while also modifying immune cell effects and thus the inflammatory microenvironment. Lastly, lysosomal granules contain hydrolases, cathepsin D and E, elastase, and lysozyme with undetermined significance (9).

### 2.3 Mechanistic pathway

Osteoarthritis (OA) is the most common cause of chronic knee pain, attributed to progressive loss and degeneration of the articular cartilage with resultant localized joint remodeling (10). Due to the synergistic combination of growth factors, cytokines, and autologous cells, PRP can promote tissue healing in a variety of mechanisms. Platelet-secreted growth factors can bind to local tissue cells, promoting cell growth and proliferation through enzymatic signaling as well as epigenetic modification. These modifications can decrease the expression of collagenases and inflammatory mediators. By inhibiting tissue breakdown and allowing replacement of damaged tissue with healthy tissue, this ultimately leads to tissue repair and regeneration.

Many of these bioactive molecules have the potential to modify inflammation at the cellular level. This occurs due to intercellular signaling, stimulating chemotaxis and mitogenesis of fibroblasts, macrophages, and neutrophils to the area, resulting in increased angiogenesis and collagen and extracellular matrix (ECM) synthesis. Additionally, histamine and serotonin released from dense granules alter capillary permeability, increasing the ability of macrophages and neutrophils to migrate to the target site. As inflammation and its downstream effects contribute significantly to the development and progression of osteoarthritis, modification of these pathways can not only slow the progression of disease but allows for local tissue healing and restoration of joint homeostasis.

In recent studies, PRP has been shown to stimulate expression of hyaluronan synthase-2, resulting in large increases in endogenous hyaluronic acid (HA) production (6). This results in similar but greater effects as exogenous injections of HA into the joint, restoring hydration of the ECM as well as restoring viscoelasticity of the synovial fluid (11).

## 3 Preparation and administration techniques

Preparation of PRP occurs via centrifugation of venous blood that then yields a concentrate of platelets and growth factors (5). There are various centrifugation protocols currently being employed and this step heavily influences the quality and concentration of the final product (2). Preparation methods most commonly include a single-spin or a double-spin protocol. One review noted 36% of studies used a single-spin protocol, while 41.3% of studies used a double-spin protocol and 22.7% of studies did not report anything (2). Another variable in the preparation process is the relative centrifugal force (RCF). RCF can be reported in various units, which vary per study protocol. When RCF is labeled in rotations per minute (RPM), it does not account for variations in rotor radius and is considered less accurate. The scientific standard of reporting of RPM is in units of gravity (g), of which only a small portion of studies at 6.7% use and is vital for reproducibility (2). Platelet activation methods can also be used. Common compounds include calcium chloride or thrombin. The use of these additives can improve growth factor release and assists with overall therapeutic outcomes (2).

Commercial PRP separation kits vary widely with respect to differences in centrifugation technique, platelet concentration and presence of leukocytes. Currently there is no universally accepted standard for PRP preparation. Different systems, such as GPS or Magellan, have differing platelet counts that range from 30% to 85% of available platelets (2). Standardization protocols seen in automated systems such as the Arthrex Angel result in higher platelet concentrations and improved clinical outcomes compared to the non-standardized methods.

Injection technique also varies for the administration of PRP for knee pain. The pathology often determines the location of where the injectate will be placed. Intra-articular injections are the standard clinical approach for osteoarthritis (11). The PRP delivery is injected directly into the joint cavity to modulate inflammation and promote cartilage repair. For musculoskeletal injections including tendinopathies, tendinitis and ligament injuries it is more common to employ the intra-tendinous or peri-ligamentous techniques (2).

Administration of PRP most commonly performed using ultrasound guidance or landmark guided techniques. The use of ultrasound guidance is recommended to accurately deliver the treatment to the correct region while also improving efficiency of the procedure (1). Information regarding the use of ultrasound has been under-reported in systematic reviews, and some analyses revealed that only a small sample size of RCTs explicitly stated that ultrasound guidance was used (1).

Standardization issues present challenges in creating universally accepted consensus statements regarding injectate material, frequency, timing and centrifugation force. It has been suggested that a PRP concentration of platelets of 1.0 × 10^6^ plt/uL appears to be optimal in respect to the blood-decree law and the PRP/media ratio (5). Other studies have investigated a dose-response relationship when platelet concentrations up to five times the baseline led to significantly elevated growth factor levels (2). Injectate volume also is an important factor to consider. Average volume injected ranged from 4.19 mL to 6.7 mL (1). Frequency and protocol variability of timing between injections also varies greatly. There is no standardized agreement for the number of injections and when they should be completed. Protocols range from a single injection to up to five injections for a treatment course (1). When multiple injections are completed, there is no industry standard set interval of time. The gaps commonly range from two weeks to one month and are often adjusted for patient specific factors including age, BMI and baseline blood counts (1).

**Figure 1:**
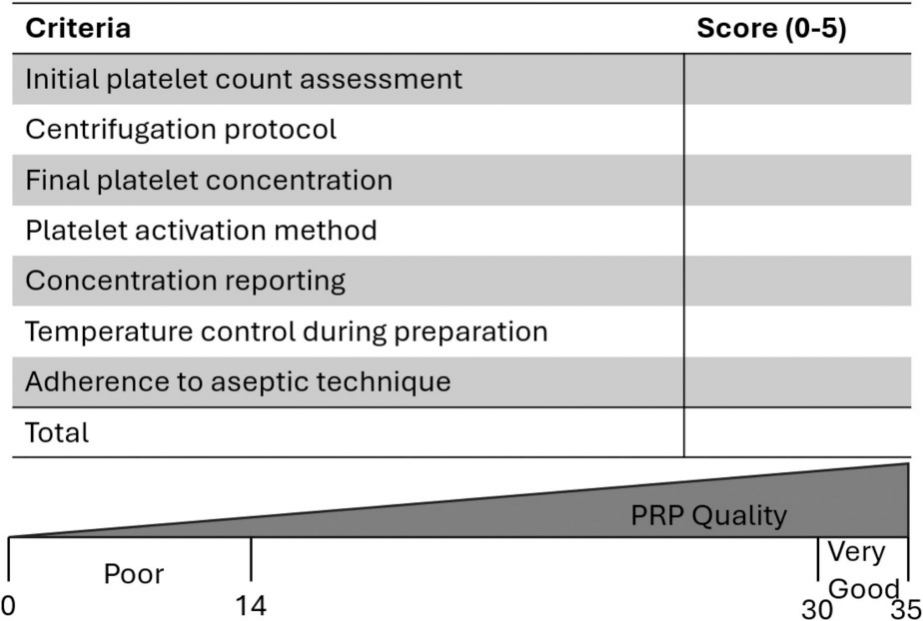
William-Eqram scoring system for PRP quality reporting (WESS-PQR) scale. Rating scale of PRP preparation quality ranging from 0 to 35. Scores of 0–14 indicate low PRP quality while 30–35 indicate very good PRP quality.

One proposed solution to combat the many significant inconsistencies in PRP preparation and reporting is the William-Eqram Scoring System for PRP Quality Reporting (WESS-PQR) (2). This scoring system is composed of seven criteria: initial platelet count assessment, centrifugation protocol, final platelet concentration, platelet activation method, growth factor concentration reporting, temperature control during preparation and adherence to aseptic techniques (2). Each category is scored on a scale from 0 to 5, with a 5 being the best or highest adherence to quality standards. The total score ranges from 0 to 35 points with 5 scoring categories that are labeled very poor adherence with significant risk of suboptimal PRP quality (0–14 points) to very good adherence to evidence based protocols (30–35 points; Figure 1).

## 4 Clinical evidence

Clinical research on intra-articular platelet-rich plasma injections for knee osteoarthritis has rapidly expanded in the past decade. Multiple randomized controlled trials, systematic reviews, and meta-analyses have been published in the past few years evaluating the safety and efficacy of this treatment. The evidence generally supports PRP's potential for improving pain and function in patients with mild-to-moderate knee osteoarthritis, though heterogeneity in study design, PRP preparation, and outcomes limits definitive conclusions.

Systematic summaries suggest that PRP's effects are maintained up to 12 months post-injection and support its usefulness in mild-to-moderate osteoarthritis (1). Several comprehensive reviews have found that PRP confers clinically relevant benefits in knee osteoarthritis compared with placebo or other injectables. A large meta-analysis reported that high-platelet concentration PRP provided significant pain relief and functional improvement at 3, 6, and 12-month follow-ups compared to placebo, with greater improvements seen in the high-platelet concentration PRP subgroup than in low-platelet concentration formulations (12). Primary RCTs included in systematic reviews show improvements in outcomes after PRP injections with patients typically experiencing significant improvements in pain relief and self-reported function improvement (WOMAC scores), over follow-up periods ranging from 3 months to 12 months (13). These findings align with meta-analysis studies that indicate PRP provides clinically meaningful benefits (1).

Pooled analyses show that PRP often results in better outcomes than hyaluronic acid (HA) injections, including higher odds of patient-reported symptom relief and achievement of minimal clinically important difference (MCID) thresholds for pain reductions (14). A 2025 meta-analsis comparing PRP + HA combination therapy vs. PRP alone found that combined treatment led to greater reductions in pain and improved functional scores with a low incidence of adverse events (11). However, some individual studies suggest that early differences between PRP and HA may be modest, with more consistent divergence emerging over longer-term follow-up (15).

The available evidence also highlights important nuances: studies differ in PRP composition (leukocyte-poor vs. leukocyte-rich), number of injections, and frequency of treatments. These variables can influence patient outcomes. Despite supportive evidence, heterogeneity among studies presents challenges for interpretation. Variations in PRP preparation (platelet concentration, leukocyte content), injection protocols, patient selection (e.g., OA severity), and outcome measures contribute to inconsistent findings across trials. Further, the substantial cost of performing rigorous large RCT's to evaluate for safety and efficacy limit the available evidence for PRP compared to studies seen in pharmaceuticals where studies are often funded through industry. Limitations in available literature makes it difficult for scientific societies to provide thorough guidelines. Despite this, some clinical guidelines have been published and remain cautious due to these inconsistencies and call for standardized protocols to improve comparability of results (15).

In summary, clinical evidence suggests that PRP injections can provide meaningful pain relief and functional improvement in patients with knee OA, particularly when high-platelet preparations are used and outcomes are assessed over 6–12 months. Meta-analyses show advantages over placebo and often over HA, though the variability in study methodologies underscores the need for standardized approaches and more high-quality RCTs to define optimal protocols and patient selection criteria.

## 5 Safety, cost, and practical considerations

When determining the overall clinical utility of PRP injections for knee pathology, clinicians must balance not only efficacy but also safety, economic burden, and real-world feasibility. Patient selection, informed consent, and shared decision-making are factors that must be considered in therapy utilization.

### 5.1 Safety profile

Overall, PRP injections are generally considered safe, largely because the product is autologous, which leads to minimized risks of allergic reactions or immunogenicity (16). Commonly reported adverse events are mild and self-limited, including transient pain at the injection site, swelling, and rash (1). Serious complications are rare but have been reported in isolated cases in the literature.

However, meta-analytic evidence suggests that PRP injections may carry a higher overall complication rate compared with placebo. A recent pooled analysis found complication rates of approximately 18.7% in PRP groups vs. 9.1% in control groups, with a number needed to harm (NNH) of 11; although most of these complications were mild to moderate and self-resolving (17).

When compared to other intra-articular injectables such as corticosteroids and hyaluronic acid, PRP does not appear to increase serious adverse events, with similar rates of minor complications reported across these treatments in randomized trials (17). Despite this similar safety profile when compared to other injectable therapies, individual factors such as advanced age or higher body mass index may be associated with increased susceptibility to local adverse reactions, highlighting the importance of patient screening and counseling.

### 5.2 Cost and insurance considerations

A major practical limitation of PRP therapies is cost, which is highly variable and often covered out-of-pocket by patients. The average annual costs for PRP injections ranged anywhere from $711.65 for ankles to $1,711.63 for hips; and by 2019 average PRP injection costs for each area totaled around $1,000 (18). PRP is often considered experimental or investigational by insurers, therefore coverage is uncommon and patients frequently must pay directly.

From a cost-effectiveness standpoint, analyses have produced mixed findings. Some decision models indicate that PRP can be cost-effective relative to conservative comparators such as hyaluronic acid injections when assessing quality-adjusted life-years (QALYs) at standard willingness-to-pay thresholds, with incremental cost-effectiveness ratios (ICERs) that remain below common U.S. benchmarks (∼$50,000/QALY) (19). However, other models comparing PRP to definitive surgical options like total knee arthroplasty (TKA) found that PRP was not cost-effective, due to uncertain long-term efficacy rather than injection cost alone (20).

### 5.3 Practical considerations for clinical use

#### 5.3.1 Patient selection and counseling

Since PRP injections require patient investment (both financial and procedural), appropriate selection criteria are crucial. Candidates often include individuals with mild-to-moderate osteoarthritis or those seeking to delay surgical interventions. Clear discussion of uncertainties in expected benefit, potential costs, and risk profile is essential in informed consent.

#### 5.3.2 Preparation and standardization challenges

Studies have highlighted significant heterogeneity in PRP preparation protocols (e.g., platelet concentration, leukocyte content, centrifugation methods), which complicates comparisons across clinical settings and may impact outcomes and adverse event rates (5). Lack of standardization also contributes to variability in cost and clinical effectiveness.

#### 5.3.3 Insurance and accessibility

Due to most insurance plans lacking coverage of PRP injections, access may be limited for patients without sufficient financial resources or healthcare savings vehicles. This has practical implications for equity of care and may influence decisions regarding trial of PRP vs. other covered therapies.

## 6 Controversies and future directions

The current climate of PRP use for knee pain has been navigating a transition from experimental use to more frequent clinical application. There have been various debates regarding consistency of use along with continued emergence of other complex biological combinations. As discussed above, a large variability exists in PRP preparation, administration and reporting. This creates a discrepancy in replication of clinical outcomes. Over 50% of RCTs failed to report baseline platelet counts and 68% failed to report the final concentration that was injected (2). Under reporting creates difficulty in the process of creating dose response relationships between platelet concentration and relief from the injectate. The William-Eqram Scoring System for PRP Quality Reporting (WESS-PQR) was proposed to address standardization issues within PRP research (2). This framework will ideally serve as a valuable resource for patient care and research efforts alike to promote consistency and reliability in the use of PRP.

Meta analyses have compared multiple treatments for knee osteoarthritis and PRP consistently ranks in the top three monotherapies for functional improvement and pain relief (1). It often outperforms hyaluronic acid (HA), corticosteroids (CS) and saline. Stem cell therapies like bone marrow aspirate concentrate (BMAC) or adipose derived stromal cells (ADSCs) are more complex compared to PRP. Papers have suggested that PRP may be more comprehensive due to the alpha granules containing bioactive protein and growth factors that stimulate tissue regeneration compared to only providing lubricant (11).

Although PRP has been efficacious, future research has been moving toward combination therapy and other “bio-functionalized approaches”. A promising direction has been the combination of PRP and HA. A recent meta-analysis noted that PRP + HA is clinically safer and more effective when compared to PRP alone. This combination has been shown to provide improved pain relief while decreasing the risk of common adverse events (11). There is a need for further mechanistic studies to evaluate the impact of temperature control during preparation and to investigate the role of leukocytes within the injectate. Currently there is a large lack of data regarding the efficacy of PRP beyond one year. Further randomized controlled trials are needed to help determine if PRP can actually halt the disease or if it is purely for symptomatic relief (1).

## 7 Conclusion

PRP injections offer a minimally invasive treatment option for select patients with knee pathology, particularly those with mild to moderate osteoarthritis. The proposed biologic mechanisms and growing clinical evidence support PRP's potential to improve pain and function, though outcomes remain inconsistent due to variability in preparation methods, injection protocols, and patient selection. Existing data suggest a favorable safety profile and possible cost-effectiveness in appropriately selected populations; however, lack of standardization, limited insurance coverage, and uncertainty regarding long-term disease modification remain significant limitations. Future studies should emphasize standardized PRP classifications, well-designed comparative trials, and long-term outcome data to better define its clinical role. With further refinement and evidence, PRP serves as a valuable adjunct in the nonoperative management of knee pathology.

## References

[1] Mende E Love RJ Young JL. A comprehensive summary of the meta-analyses and systematic reviews on platelet-rich plasma therapies for knee osteoarthritis. Mil Med. (2024) 189(11–12):e2347–56. 10.1093/milmed/usae022 38421752

[2] Rahman E Rao P Abu-Farsakh HN Thonse C Ali I Upton AE Systematic review of platelet-rich plasma in medical and surgical specialties: quality, evaluation, evidence, and enforcement. J Clin Med. (2024) 13(15):4571. 10.3390/jcm13154571 39124838 PMC11313071

[3] Felipone WK Mambro LD Ranieri BR Ivanov GZ Meves R Martins L The controlled release of platelet-rich plasma–loaded alginate repairs muscle damage with less fibrosis. Am J Sports Med. (2025) 53(5):1152–63. 10.1177/03635465251321108 39994839

[4] Han J Ding Z Huang J Zhang Y Ding Y. Pure platelet-rich plasma delays intervertebral disc degeneration by activating SIRT1-mediated autophagy in nucleus pulposus cells. J Orthop Surg Res. (2025) 20(1):786. 10.1186/s13018-025-06205-0 40846953 PMC12372246

[5] Gentile P Garcovich S. Systematic review—the potential implications of different platelet-rich plasma (PRP) concentrations in regenerative medicine for tissue repair. Int J Mol Sci. (2020) 21(16):1–22. 10.3390/ijms21165702 PMC7460839 32784862

[6] Gilbertie JM Long JM Schubert AG Berglund AK Schaer TP Schnabel LV. Pooled platelet-rich plasma lysate therapy increases synoviocyte proliferation and hyaluronic acid production while protecting chondrocytes from synoviocyte-derived inflammatory mediators. Front Vet Sci. (2018) 5:150. 10.3389/fvets.2018.00150 30023361 PMC6039577

[7] Lana JF Huber SC Purita J Tambeli CH Santos GS Paulus C Leukocyte-rich PRP versus leukocyte-poor PRP—the role of monocyte/macrophage function in the healing cascade. J Clin Orthop Trauma. (2019) 10:S7–S12. 10.1016/j.jcot.2019.05.008 31700202 PMC6823808

[8] Yuan C Ang SP Hasoon JJ Tolba R Ruan QZ Lam CM Dual-action regenerative therapies: regeneration and antimicrobial effects of platelet- and marrow-derived biologics. Biomedicines. (2025) 13(11):2832. 10.3390/biomedicines13112832 41301922 PMC12650291

[9] Szwedowski D Szczepanek J Paczesny Ł Zabrzyński J Gagat M Mobasheri A The effect of platelet-rich plasma on the intra-articular microenvironment in knee osteoarthritis. Int J Mol Sci. (2021) 22(11):5492. 10.3390/ijms22115492 34071037 PMC8197096

[10] Vanneste T Belba A Oei GTML Emans P Fonkoue L Kallewaard JW 9. Chronic knee pain. Pain Pract. (2025) 25(1):e13408. 10.1111/papr.13408 39219017 PMC11680467

[11] Du D Liang Y. A meta-analysis and systematic review of the clinical efficacy and safety of platelet-rich plasma combined with hyaluronic acid (PRP + HA) versus PRP monotherapy for knee osteoarthritis (KOA). J Orthop Surg Res. (2025) 20(1):57. 10.1186/s13018-024-05429-w 39819683 PMC11740359

[12] Bensa A Previtali D Sangiorgio A Boffa A Salerno M Filardo G. PRP injections for the treatment of knee osteoarthritis: the improvement is clinically significant and influenced by platelet concentration: a meta-analysis of randomized controlled trials. Am J Sports Med. (2025) 53(3):745–54. 10.1177/03635465241246524 39751394 PMC11874499

[13] Shen L Yuan T Chen S Xie X Zhang C. The temporal effect of platelet-rich plasma on pain and physical function in the treatment of knee osteoarthritis: systematic review and meta-analysis of randomized controlled trials. J Orthop Surg Res. (2017) 12(1):16. 10.1186/s13018-017-0521-3 28115016 PMC5260061

[14] Oeding JF Varady NH Fearington FW Pareek A Strickland SM Nwachukwu BU Platelet-rich plasma versus alternative injections for osteoarthritis of the knee: a systematic review and statistical fragility index–based meta-analysis of randomized controlled trials. Am J Sports Med. (2024) 52(12):3147–60. 10.1177/03635465231224463 38420745

[15] Glinkowski WM Gut G Śladowski D. Platelet-rich plasma for knee osteoarthritis: a comprehensive narrative review of the mechanisms, preparation protocols, and clinical evidence. J Clin Med. (2025) 14(11):3983. 10.3390/jcm14113983 40507744 PMC12156035

[16] Latalski M Walczyk A Fatyga M Rutz E Szponder T Bielecki T Allergic reaction to platelet-rich plasma (PRP). Medicine (Baltimore). (2019) 98(10):e14702. 10.1097/MD.0000000000014702 30855461 PMC6417490

[17] Fucaloro SP Bragg J Berhane M Mulvey M Krivicich L Zink T Complications of platelet-rich plasma injection for knee osteoarthritis are similar to those of corticosteroids and hyaluronic acid but are significantly greater than those of placebo injections: a meta-analysis of randomized controlled trials. Arthroscopy. (2025) 41(11):4789–803. 10.1016/j.arthro.2025.05.018 40409439

[18] Magruder ML Caughey S Gordon AM Capotosto S Rodeo SA. Trends in utilization, demographics, and costs of platelet-rich plasma injections: a ten-year nationwide investigation. Phys Sportsmed. (2024) 52(1):89–97. 10.1080/00913847.2023.2178816 36755520

[19] Samuelson EM Ebel JA Reynolds SB Arnold RM Brown DE. The cost-effectiveness of platelet-rich plasma compared with hyaluronic acid injections for the treatment of knee osteoarthritis. Arthroscopy. (2020) 36(12):3072–8. 10.1016/j.arthro.2020.07.027 32721546

[20] Rajan PV Ng MK Klika A Kamath AF Muschler GF Higuera CA The cost-effectiveness of platelet-rich plasma injections for knee osteoarthritis: a markov decision analysis. J Bone Joint Surg Am. (2020) 102(18):E104. 10.2106/JBJS.19.01446 32453118

